# Self-supervised pre-training for joint optic disc and cup segmentation via attention-aware network

**DOI:** 10.1186/s12886-024-03376-y

**Published:** 2024-03-04

**Authors:** Zhiwang Zhou, Yuanchang Zheng, Xiaoyu Zhou, Jie Yu, Shangjie Rong

**Affiliations:** 1 Institute for Advanced Study, Nanchang University, Nanchang, 330031 China; 2 Institute of Science and Technology, Waseda University, Tokyo, 63-8001 Japan; 3 School of Transportation Engineering, Tongji University, Shanghai, 200000 China; 4 School of Electrical Automation and Information Engineering, Tianjin University, Tianjin, 300000 China; 5 School of Mathematical Sciences, Xiamen University, Xiamen, 361000 China

**Keywords:** Deep learning, Optic disc and cup segmentation, Medical image processing

## Abstract

Image segmentation is a fundamental task in deep learning, which is able to analyse the essence of the images for further development. However, for the supervised learning segmentation method, collecting pixel-level labels is very time-consuming and labour-intensive. In the medical image processing area for optic disc and cup segmentation, we consider there are two challenging problems that remain unsolved. One is how to design an efficient network to capture the global field of the medical image and execute fast in real applications. The other is how to train the deep segmentation network using a few training data due to some medical privacy issues. In this paper, to conquer such issues, we first design a novel attention-aware segmentation model equipped with the multi-scale attention module in the pyramid structure-like encoder-decoder network, which can efficiently learn the global semantics and the long-range dependencies of the input images. Furthermore, we also inject the prior knowledge that the optic cup lies inside the optic disc by a novel loss function. Then, we propose a self-supervised contrastive learning method for optic disc and cup segmentation. The unsupervised feature representation is learned by matching an encoded query to a dictionary of encoded keys using a contrastive technique. Finetuning the pre-trained model using the proposed loss function can help achieve good performance for the task. To validate the effectiveness of the proposed method, extensive systemic evaluations on different public challenging optic disc and cup benchmarks, including DRISHTI-GS and REFUGE datasets demonstrate the superiority of the proposed method, which can achieve new state-of-the-art performance approaching 0.9801 and 0.9087 *F*1 score respectively while gaining 0.9657 $DC_{disc}$ and 0.8976 $DC_{cup}$. The code will be made publicly available.

## Introduction

Glaucoma is the leading cause of irreversible vision damage, and it is reported that the number of glaucoma patients will increase to 110 million worldwide by 2040 [1, 2]. Glaucoma progresses silently without earlier noticeable symptoms. To prevent permanent vision loss, early treatment is extremely important. Recently, there have been three common diagnostic techniques for glaucoma including optic nerve head assessment [3], function-based visual field examination [4, 5], and intraocular pressure (IOP) assessment [6, 7]. Among these, some manual assessment methods of intraocular pressure measurement have not been widely used due to the differences in the human and equipment resources of each hospital. At the same time, these manual assessment methods consume a lot of manpower and are not conducive to large-scale pathological screening in hospitals, which may hinder their application in the real-life world.

**Fig. 1:**
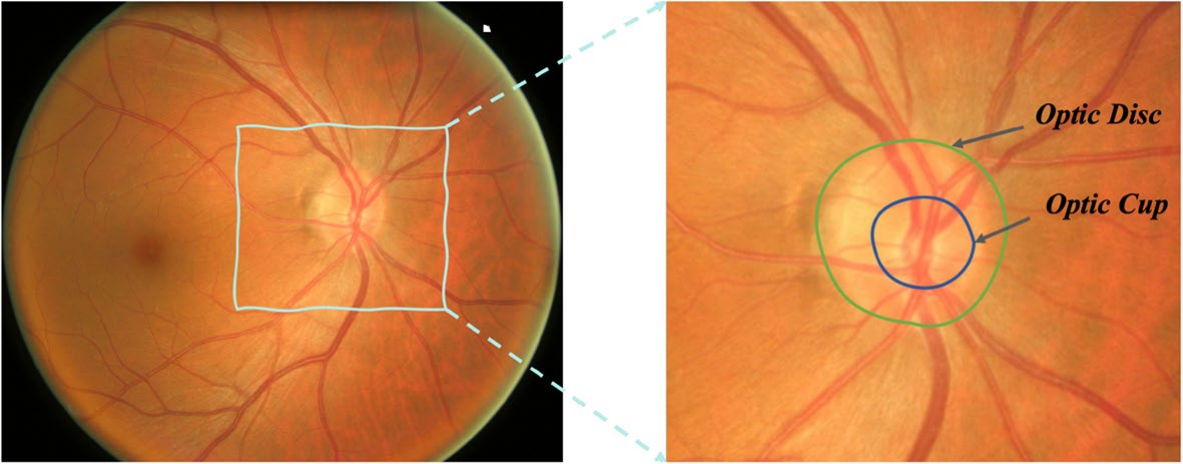
Visualization of the retinal fundus images and the corresponding OD and OC images

To this end, the automatic retinal fundus photography strategy [8, 9] using deep neural networks becomes popular, which can help doctors to screen glaucoma. As shown in Fig. 1, the retinal fundus image shoots the main structure of the fundus including the optic disc (OD) and optic cup (OC). The vertical cup-to-disc ratio (CDR) can be calculated by the comparison of the diameter of the cup-to-disc. The normal CDR is 0.3 to 0.4. A larger CDR may indicate glaucoma. The accurate CDR can be calculated from the segmented optic disc and cup area [10].

Currently, deep learning methods [11] show great performance towards the accurate optic disc and cup segmentation. The most prominent architecture is U-Net [12], which performs skip connections to fuse multi-level information. Later, M-Net [13] further improves the performance by injecting the domain-specific knowledge that the optic cup lies in the optic disc and adds side-output layers to acquire more supervision. JointRCNN [14] explores joint OD and OC segmentation with a disc attention module and makes full use of the prior knowledge that the optic disc and cup are approximately ellipses. PM-Net [15] performs OD and OC detection and also utilises the prior knowledge that the optic cup lies in the optic disc. Afterwards, Yin et al. [16] inject a guided filter into U-Net to restore the structure information loss caused by down-sampling operations. The domain-specific knowledge helps to increase the performance. Some algorithms [17, 18] try to adopt GAN [19] to assist in enhancing segmentation performance. Recently, some of the up-to-date networks [20, 21] utilize vision transformer [22, 23] to conduct medical image segmentation and achieve state-of-the-art performance.

However, two challenging questions remain on the optic disc and cup segmentation task: (1) How to design an effective network to capture the global information of the input images and enjoy fast execution; (2) How to solve the problem that optic disc and cup training samples are not sufficient enough. For the first issue, most previous works will explore non-local skill [24] to capture the semantics of the medical images. However, excessive convolution operations complicate the calculation, which is prone to overfitting. Besides, some of the token-based transformer methods are too large, which take a lot of computing resources and execute slowly, which is not practical in a real medical environment. For the second point, collecting medical images (e.g. optic disc and cup images) is much more difficult than in the common computer vision field data (i.e. the public COCO [25] and PASCAL [26] segmentation datasets can be widely collected on the Internet) due to some pathological privacy issues. Therefore, training the deep learning networks especially some transformer or GAN-based networks may achieve unsatisfactory results when training data is rare.

In this paper, to tackle the above-mentioned issues, we propose a novel attention-aware segmentation model equipped with the multi-scale attention module in the pyramid structure-like encoder-decoder network, which can efficiently learn the global semantics and the long-range dependencies of the input images. The proposed multi-scale attention module is different from the traditional attention mechanism in transformer [22], we design a more powerful multi-scale nearest neighbour semantic pixel matching operation to enable the network to capture more useful visual hints. Besides, different from some previous methods that require multi-stages [27] for segmentation, our framework is a one-stage network, which does not need first to crop the key region and then segment the image. Furthermore, considering that the scarcity of medical imaging images leads to instability in training deep network models, we designed a new self-supervised contrastive learning training paradigm, which can learn the discriminative representation of the image in an unsupervised manner. Meanwhile, we also proposed a novel loss function to make use of this knowledge by constraining the subtraction of the optic cup from the optic disc in the optic rim.

To demonstrate the effectiveness of the proposed method, extensive systemic evaluations on different public challenging optic disc and cup benchmarks including DRISHTI-GS and REFUGE datasets reveal the superiority of the proposed method, which can achieve new state-of-the-art performance. Our main contributions are summarized as follows:

- We experimentally analyze unsolved challenges in optic disc and cup segmentation tasks, and we take the early step to explore self-supervised contrastive learning to tackle the drawbacks in the medical image field.
- We propose a brand new attention-aware segmentation model equipped with the multi-scale attention module, which explores nearest neighbour semantic pixel matching operation to enable the network to capture more useful visual cues for optic disc and cup segmentation.
- We design a novel loss function to make use of the knowledge by constraining the subtraction of the optic cup from the optic disc in the optic rim, which can help to enhance the learning representation.
- Extensive experimental results conducted on different challenging benchmarks validate the effectiveness of the proposed network and training paradigm, which can achieve new state-of-the-art performance.

## Related work

In this section, we will provide a brief overview of different types of existing traditional and medical image segmentation methods. Specifically, we will summarize the ordinary scene image segmentation methods based on CNNs or transformers, and then review the expansions of these methods in the medical image domain. Finally, we will discuss the self-supervised training methods.

**Non-Learning-Based Image Segmentation**: Image segmentation is a crucial preprocessing for image recognition and computer vision. Conventional image segmentation usually means traditional semantic segmentation. Image segmentation in this period (about 2010), due to limited computer computing power, could only process some grayscale images in the early days, and later could process rgb images. The segmentation in this period mainly depends on extracting low-level features of images and then segmenting them, some methods have emerged: Ostu [28], FCM [29], watershed [30], N-Cut [31], etc. Subsequently, with the improvement of computing power, people began to consider obtaining semantic segmentation of images. The semantics here are currently low-level semantics, which mainly refers to the categories of segmented objects. At this stage (probably from 2010 to 2015), people considered using machine Learning methods for image semantic segmentation. With the emergence of FCN [32], deep learning officially enters the field of image semantic segmentation.

**Image segmentation based on CNNs**: Image segmentation is a vital branch in the field of deep learning, which can help analyze the pixel-level content of images. The first step of traditional image segmentation usually need to collect a large amount of data (i.e. collect the images from the Internet), and then requires enormous annotations to train a strong network for satisfactory performance. Long et al. [32] proposed fully convolutional networks (FCNs), which enjoys advantageous in end-to-end dense representation modeling, laying the foundation for modern semantic segmentation algorithms. However, FCNs suffer from the limited visual context with local receptive fields of the convolutional operations. Later, DeepLab [33–35] explores new solution by enlarging receptive fields with dilated operation and spatial pyramid pooling. Moreover, scholars try to design different pyramid-like structure network [36, 37] for multi-scale learning. Some other researchers utilize U-Net [12] like structure and devise many promising encoder-decoder network [38, 39] solutions. Furthermore, many existing works adopt auxiliary information like boundary clues [40, 41] and optical flow [23] hints to boost performance. Recently, many cutting-edge semantic segmentation methods inject neural attention [24, 42–45] for improving the extracted semantic features. As for medical image segmentation, U-Net series can help achieve competitive performance. Edupuganti et al. [46] adopts an end-to-end encoder-decoder network to segment optic disc and cup with the edge loss function. Shankaranarayana et al. [47] utilizes FCN network with adversarial training for OD and OC joint segmentation. Later Fu et al. [13] proposes M-Net with multi-label strategy for segmentation. More recently, some other variants networks like U-Net++ [48], U-Net3+ [49] and DenseUNet [50] also shows acceptable performance in medical image segmentation. In MDC-Net [51], multi-scale dilated convolution is adopted to increase the receptive field of the model and multiple residual connections are used to utilize feature information from different scales. Zhu et al. [52] designed a network consisting a down-sampling path extracting the features and an up-sampling path restoring the down-sampled features. The features are automatically extracted from the images through the convolutional operators during the down-sampling procedure. Besides, some other latest works [53, 54] both adopted deep- learning-based method to automaticly for pathological analysis. Nevertheless, although the mentioned methods have used variants of encoder-decoder architecture, they limit the local context encoding by convolutional layers. To this end, some researchers’ focus gradually shifts to vision transformer.

**Image segmentation based on transformer**: Recently, more and more segmentation models [55, 56] are built upon the attention vision transformer (ViT) [22] to capture the global long-range dependencies of the image pixels. Zheng et al. [57] explores ViT as backbone and utilize a standard CNN as decoder for segmentation. Swin Transformer [58] designs a variant of ViT architecture with shifted windows and equipped with a pyramid FCN decoder. Robin et al. [56] proposes a transformer encoder-decoder architecture for semantic image segmentation inspired by DETR [59]. As in medical image segmentation, TransUNet [60] designs a U-Net like transformer network to locate the image token spatial information. TransAttUnet [61] improves the U-shaped architecture segmentation network with multi-level guided attention and multi-scale skip connection. DS-TransUNet [62] adopts the Swin Transformer block [58] to both the encoder and the decoder and achieve competitive performance. Liu et al. [63] proposed a network which consists of a transformer-based branch and a convolution-based branch, and the information is exchanged between the inner layers. However, all the above-mentioned medical segmentation methods fails to take full advantage of the spatial detail information from the transformer-based network since the medical training images are insufficient, which greatly increases the difficulty of transformer network training.

**Self-supervised training methods**: In recent years, unsupervised or self-supervised learning has attracted much attention. Some previous methods design the pretext task like image colorization [64], image jigsaw complement [65, 66] and rotation prediction [67], etc. With the birth of the contrastive learning paradigm, MOCO [68] learns the feature representation using a dictionary look-up pretext task from a perspective on contrastive learning. SimpleCLR [69] learns representations by maximizing agreement between differently augmented views of the same data example via a contrastive loss in the latent space. SimpleCLR and MOCO both adopt a siamese network with contrastive learning and achieve encouraging performance. Besides, some video self-supervised learning methods focus on temporal hints for learning. Xu et al. [70] model the self-supervised learning by shuffling the video and predicting the final orders. Some other works [71, 72] pay attention to complete video playback speeds. Therefore, in this paper, we explore the self-supervised learning for optic disc and cup segmentation based on contrastive learning and explore several data augmentation methods to acquire a pre-train model for segmentation.

## Methodology

In this section, we will introduce the pipeline of the proposed network. First, the overview of the network architecture is presented, followed by a detailed description of each component. Then, we introduce the self-supervised pre-training paradigm of the proposed network. Finally, we will explicitly elaborate on loss functions to train our network.

### Network architecture

**Fig. 2:**
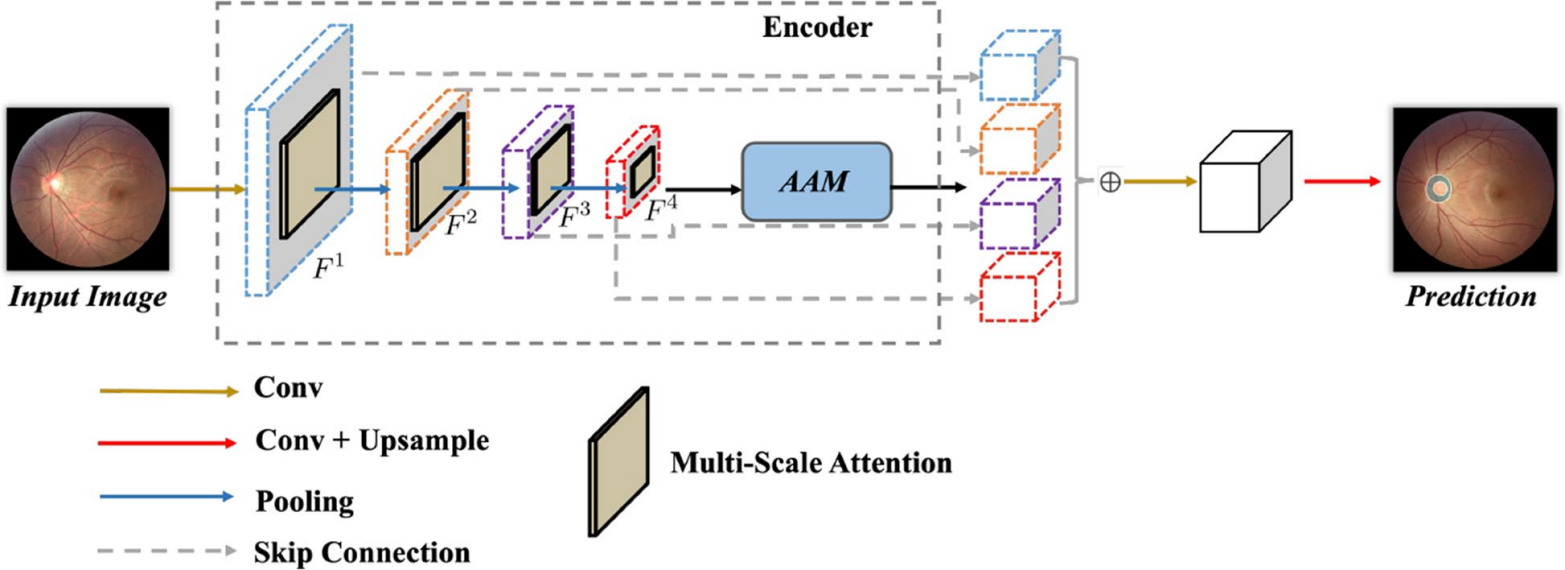
The overall architecture of the network. The given input image *I* is first fed into the encoder, yielding the multi-scale feature maps *F*. We employ the proposed multi-scale attention module followed by each convolutional layer for feature enhancement. Then, we inject the designed aggregation attention module followed by the last layer for feature fusion. The decoder is bridged behind the encoder in the pyramid-like structure for final mask prediction

As shown in Fig. 2, we depict the overall network architecture of the proposed network. Given the input image *I*, our target is to segment out the accurate mask output *O* that represents the optic disc and cup. To achieve this goal, we propose an attention-aware segmentation network, which is based on an encoder-decoder structure. Specifically, we adopt the CNN encoder (i.e. ResNet [73]) to extract the multi-scale feature map ($F_1$, $F_2$, $F_3$, $F_4$). For each layer, we add the proposed multi-scale attention module followed by the convolutional feature maps to model the global semantic hints. The aggregation attention module is followed by the last feature layer for enhancement. The overall network is in the pyramid structure-like architecture with different skip-connection. The decoder is responsible for upsampling and predicting the final masks.

**Fig. 3:**
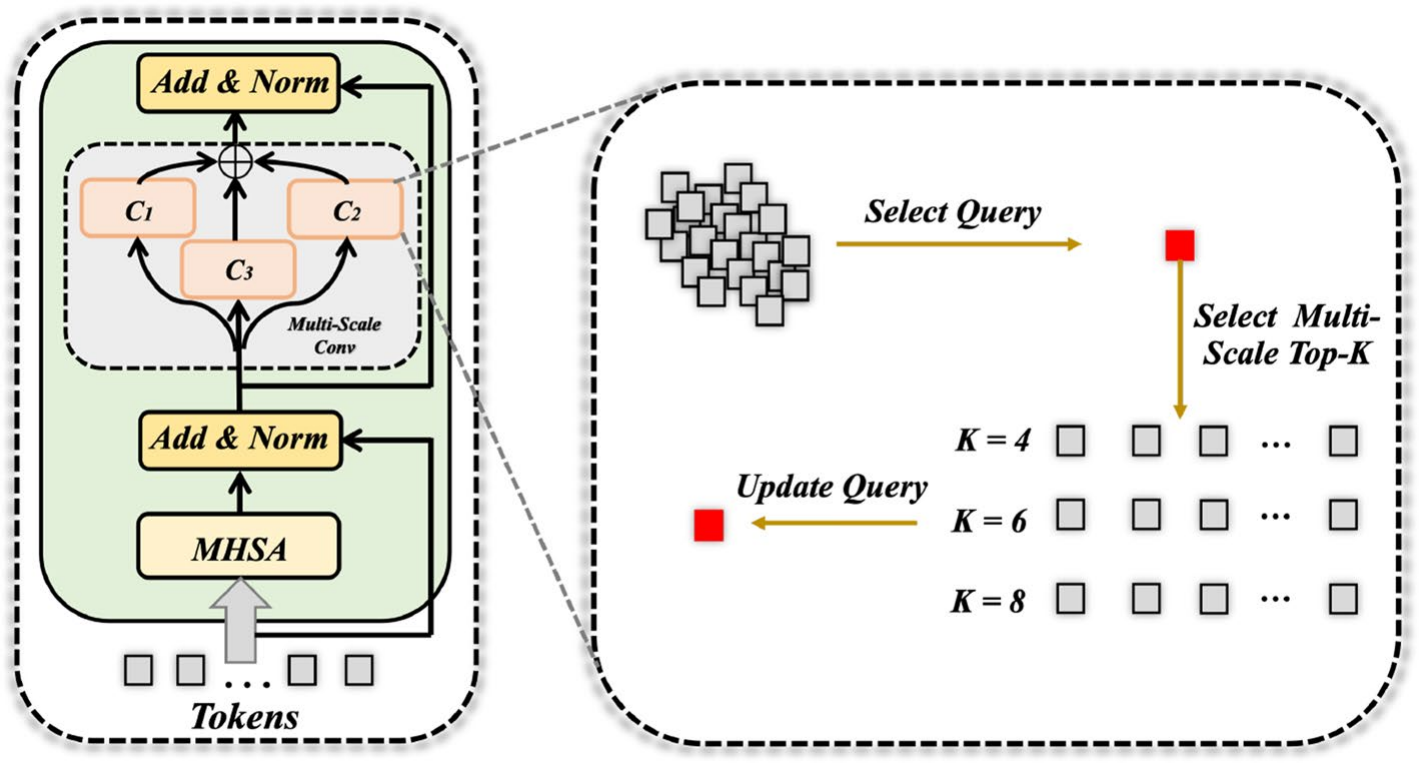
Illustration of the proposed multi-scale attention module. For each query image token pixel, it will match with its top-*K* potentially corresponding tokens. Afterwards, it will be updated by aggregating different sub-region representations using the multi-layer perceptron operation

**Multi-Scale Attention Module**: Concretely, we introduce the proposed multi-scale attention module followed by each convolutional layer for global feature modelling, as shown in Fig. 3. For simplicity, we consider ($$F_1$$, $$F_2$$, $$F_3$$, $$F_4$$) to be *F*. Specifically, we first reshape the feature map $$F \in \mathbb {R}^{B*N \times C \times h \times w}$$ (*w* and *h* denote the spatial size of the feature map, *C* is the feature dimension, *B* and *N* denote the batch size and group size, respectively) into a sequential flattened patch tokens $$X \in \mathbb {R}^{B \times C \times P}$$, where $$P = N*h*c$$. Then, we adopt the multi-head sel-attention mechanism (MHSA) for these tokens, which can be computed as follows:

1 $$\begin{aligned} X = MHSA(LN(X)) + X, \end{aligned}$$

where *LN* indicates LayerNorm [74] function.

Afterwards, different from the standard vision transformer [22] operation that directly applies linear layers after the MHSA, we propose the multi-scale attention multilayer perceptron here. Specifically, as shown in Fig. 3 right, for each query patch token, it will first select its corresponding top-*k* nearby potential tokens. Mathematically, for each token, we first use ${\ell _2}$- distance to measure the relationship between the two arbitrary patches. Since we use normalized channel features, by removing the constant, the matrix $S \in \mathbb {R}^{B*N \times Q \times Q}$ ($Q = h * w$ is the number of patches) can be formulated as:

2 $$\begin{aligned} S = {F}^{T} {F}. \end{aligned}$$

We then perform *KNN* operation on the matrix using the PyTorch built-in function (*torch*.*topk*(*S*)) to select its potentially corresponding target patches, which will produce a tensor $\hat{X} \in \mathbb {R}^{B \times C \times N \times K}$, which indicates the patches along with their top-*K* semantically related patches. Here, we chose k = 4, 6, 8. In a word, for each query token, it can match tokens of different scales that are semantically similar. When *k* becomes larger, it contains more relevant tokens. In order to update the token’s features, the multi-scale Multi-layer Perceptron (MSMLP) embedding operation is performed as:

3 $$\begin{aligned} X = \sum\limits_{i}^{k=4,6,8} \text {Max}(\underset{x \in \hat{X}}{\varphi }(x_1, x_2, ... , x_{k_i})), \ \ X \in \mathbb {R}^{B \times C \times N}, \end{aligned}$$

where $\varphi$($\cdot$) is the local feature modelling function, and we here use two 1 $\times$ 1 convolution layers followed by the ReLU activation function, and we fuse the multi-scale features by tensor addition.

Ultimately, the whole process of each multi-scale attention module can be formulated as follows:

4 $$\begin{aligned} X'&= \text {MHSA}(\text {LN}(X)) + X, \\ X&= \text {MSMLP}(\text {LN}(X')) + X', \end{aligned}$$

**Fig. 4:**
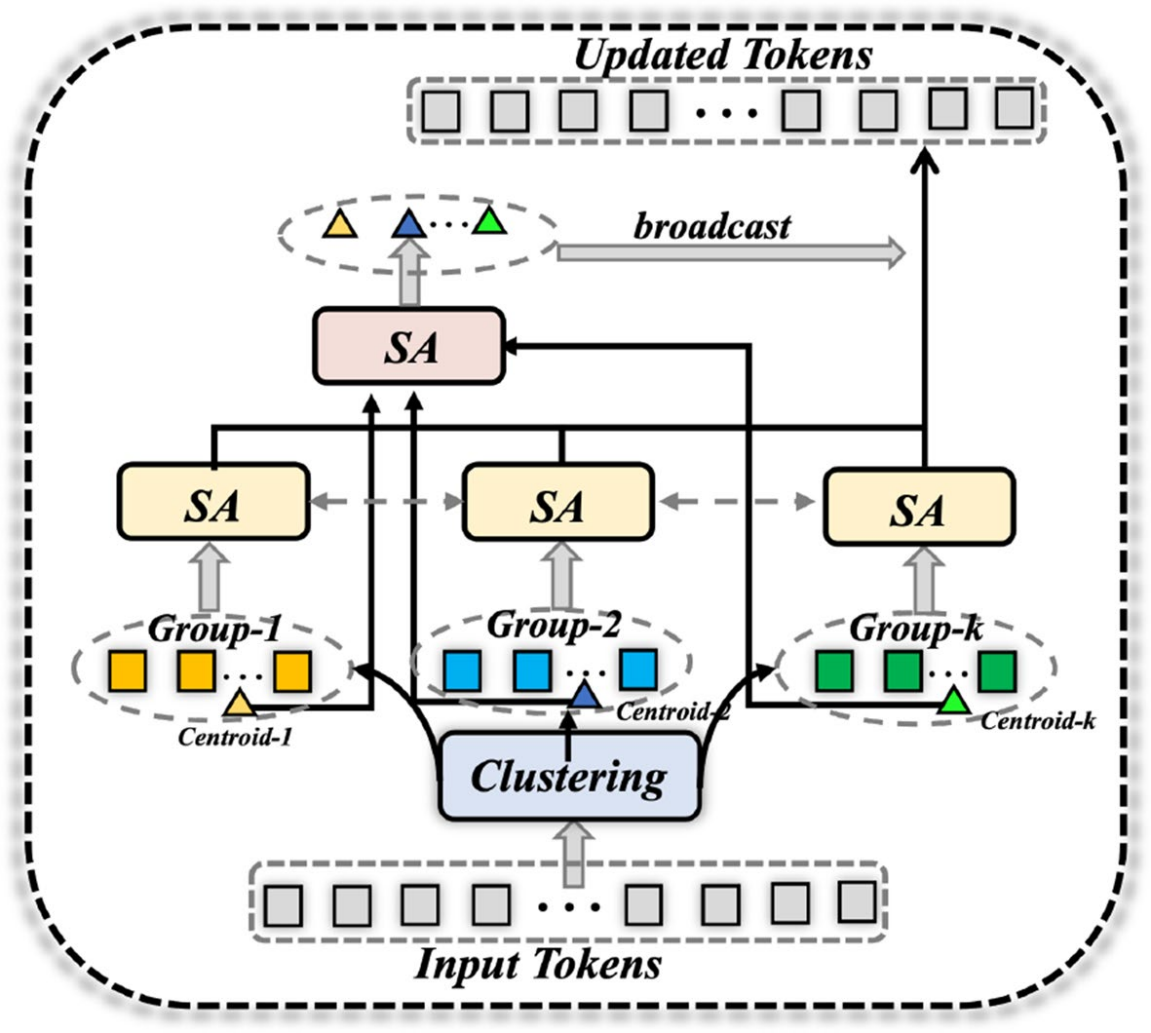
Illustration of the proposed aggregation attention module. The input tokens are first clustered into different groups. For each group, the self-attention operation is performed individually over the cluster centroid and cluster tokens. Ultimately, the updated cluster centroid and the group features are aggregated together to form a new feature vector

**Aggregation Attention Module**: Furthermore, we design an aggregation attention module following the last layer $F_4$. As shown in Fig. 4, for the input tokens, we first utilize a mini-batch k-means clustering algorithm to group query into $C=3$ clusters adaptively followed the implementation in [75]. Here, we want to cluster the image tokens into the optic disc, cup and background. Concretely, for the cluster centroid and cluster tokens, we both adopt the self-attention mechanism to update the global features as follows:

5 $$\begin{aligned} Q = {\textbf {W}}_Q X + {\textbf {B}}_Q , \ \ K = {\textbf {W}}_K X + {\textbf {B}}_K, \ \ V = {\textbf {W}}_V X + {\textbf {B}}_V, \end{aligned}$$

where ${\textbf {W}}_Q, {\textbf {W}}_K$ and ${\textbf {W}}_V$ are three learnable linear weight matrices, while ${\textbf {B}}_Q, {\textbf {B}}_K$ and ${\textbf {B}}_V$ are weight vectors. After having the *Q*, *K*, *V*, the global self-attention mechanism can be formulated as:

6 $$\begin{aligned} \text {Attention}(Q, K, V) = \text {SoftMax}(Q K^{T} / \sqrt{D}) V, \end{aligned}$$

where *D* is the feature dimension. Afterwards, the updated cluster centroid are broadcasted into the shape of the input tokens and combined with the updated group cluster tokens to produce the output.

Finally, the decoder with skip connections fuse multi-level information and produces the segmentation prediction of the optic disc and cup. However, the deep guidance network does not inject domain-specific knowledge. Motivated by the prior knowledge that the subtraction of the optic cup from the optic disc is the optic rim, we design a multi-label loss to inject this knowledge. As suggested by PM-Net [15], the multi-label head learns an independent binary classifier for each class. Furthermore, to segment a glaucoma fundus image that the OC occupies the most area of OD, the multi-label head balances the pixel number for OD and OC since the classifier is independent for OD and OC. However, the multi-label head does not make full use of the ground truth. We further add the constraint that the subjection of OC from OD is the optic rim (OR). The proposed loss function treats the segmentation problem as three binary classification problems with single label: [OD, OD^-^], [OC, OC^-^], [OD-OC, OR^-^] (^-^ represents negative examples). Then, the loss function can be defined as follows:

7 $$\begin{aligned} Loss_D&= {} g_{D,i} log p_{D,i} + (1-g_{D,i}) log (1- p_{D,i}),\\ Loss_C&= {} g_{C,i} log p_{C,i} + (1-g_{C,i}) log (1- p_{C,i}),\\ Loss_R&= {} g_{R,i} log (p_{D,i} - p_{C,i}) + (1-g_{R,i}) log (1- (p_{D,i} - p_{C,i})), \end{aligned}$$

where, $g_{R,i}$ represents the ground truth of the optic rim and can be calculated by $g_{R,i}$ = $g_{D,i}-g_{C,i}$. Finally, the total segmentation loss can be defined as follows:

8 $$\begin{aligned} Loss = \frac{1}{3} * ( Loss_D + Loss_C + Loss_R). \end{aligned}$$

Here we treat the segmentation problem as three binary classification problems. $p_{D,i}$, $p_{C,i}$ represents the predicted probability of OD and OC respectively for pixel *i*. $g_{D,i}$, $g_{C,i}$ represents the ground-truth label of pixel *i* for OD and OC respectively. We add a constraint that the predicted OD area subject the predicted OC area is close to the ground truth OR area.

### Self-supervised pretrainng

Self-supervised learning aims to learn feature representations from a large amount of unlabeled data, which is usually achieved by setting different pretext tasks and utilize easy-to-obtain automatically generated supervision. In the image domain, [64] perform image colorization pretext to establish a mapping from objects to colors that learn the potential features of the images. Some previous works [65, 66] try to solve jigsaw problems to learn the information of different patches in the images. Komodakis et al. [67] proposed a simple rotation transformation to make the network to predict different rotation degrees of the images to identify objects’ features. Later, such transformations as scaling, warping and inpainting have been applied to the latest work [76]. Leveraging the merits of contrastive learning that focus on semantic information rather than too much on pixel details, most of the current works [68, 77, 78] explored to construct positive pairs and negative pairs for feature learning.

**Fig. 5:**
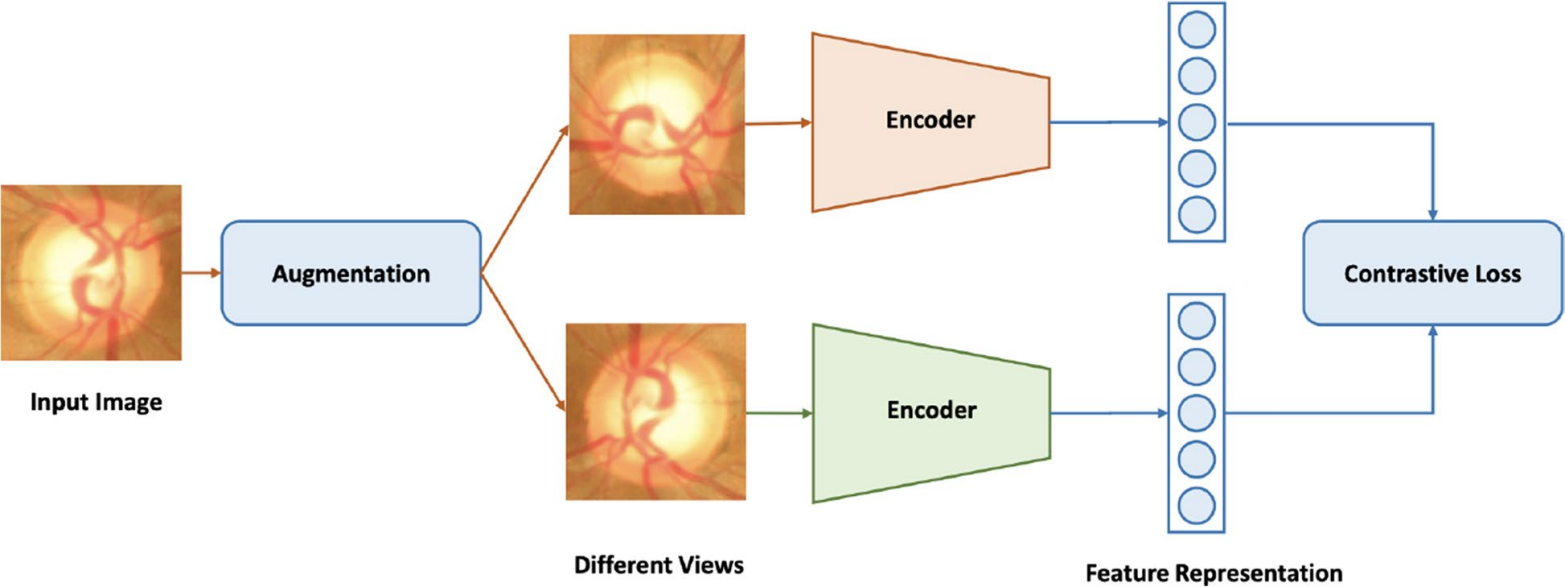
The framework of the proposed self-supervised method. An input image is augmented into two different views. Then the network learns to maximize agreement using a contrastive loss

The self-supervised framework is shown in Fig. 5. We perform two separate data augmentation operations to obtain two different views of an input image. Then, we train our network to maximize the agreement using a contrastive loss. We randomly sample a mini-batch of *N* examples and define the contrastive prediction task on pairs of augmented examples derived from the mini-batch. The two views are similar to each other and dissimilar to other pairs. The similarity is measured by the dot product. The InfoNCE loss function [79] is considered in the paper to train the network, and it is defined as follows:

9 $$\begin{aligned} L_q = -log \frac{exp(q \cdot k_+) / \tau }{\sum ^{K}_{i=0}exp(q \cdot k_i) / \tau }. \end{aligned}$$

where, *q* and $k_+$ are the positive pair. *q* and $k_i$ are *K* negative pairs. The sum is over one positive and *K* negative samples. $\tau$ denotes a temperature parameter [80].

**Fig. 6:**
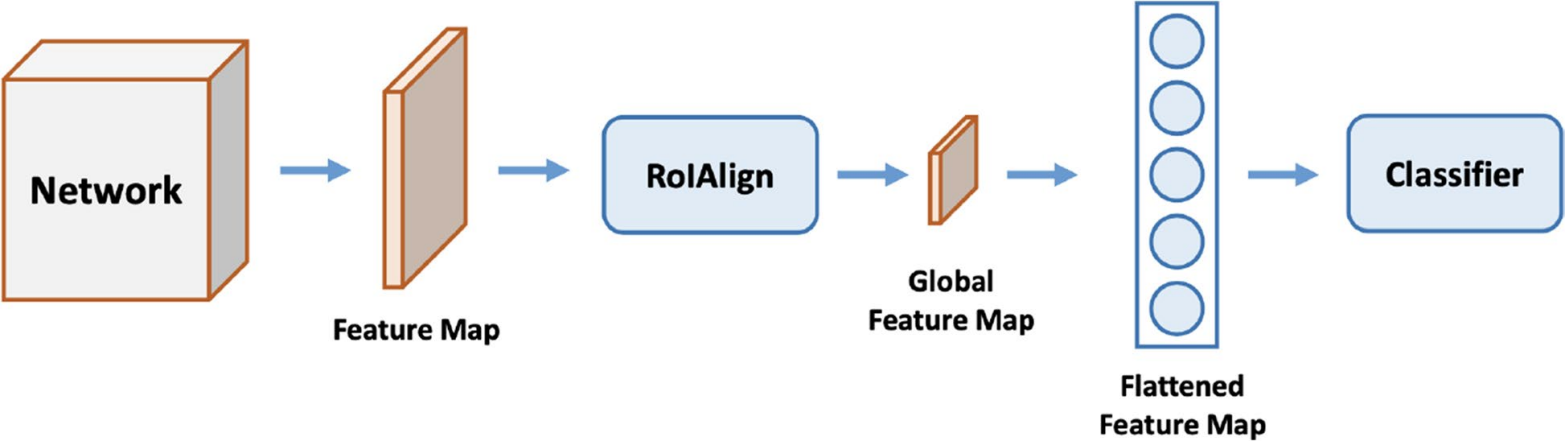
The self-supervised training head for segmentation. The input image is first encoded by the network encoder. Then RoiAlign operation is applied to obtain a smaller global feature map for efficient learning. The final fully connected layer flattens the feature for contrastive learning

Moreover, RotNet [67] trains a network to recognize the rotation transformation for unsupervised learning and motivated by this, we also apply the rotation transform augmentations. Similar to RotNet, we only rotate the image to 0^∘^, 90^∘^, 180^∘^, 270^∘^. MOCOv2 [81] states that the gaussian blur is also helpful for learning, so we also perform gaussian blur with $\sigma$ between [0, 0.5]. The sharpening operation is to sharpen images and alpha-blend the result with the original input images. When $\alpha$ = 0, only the original image is visible. When $\alpha$ = 1, only its sharpened version is visible. We also conduct $\gamma$ contrast with $\gamma$ between [0.5, 2] to augment the data. For segmentation, the output resolution is usually large (For example, 512 $\times$ 512), after the flatten operation, it will make the following fully connected layers too large to train. To solve this issue, we adopt the RoiAlign layer proposed by Mask-RCNN [82] to obtain a smaller global feature map (24 $\times$ 24). The global feature map is flattened and sent to the classifier for contrastive learning, where the entire process is shown in Fig. 6. Moreover, Table 1 shows the augmentation we used for self-supervised learning.

**Table 1:** The data augmentation used in pretraining

Augmentation	Parameters
Rotation	0^∘^, 90^∘^, 180^∘^, 270^∘^
Sharpen	$\alpha$ = [0, 1]
GammaContrast	$\gamma$ = [0.5, 2.0]
GaussianBlur	$\sigma$ = [0, 0.5]

## Experiment

In this section, we will first elaborate on the details of experiment settings including datasets, metrics and detailed implementation. We then analyze the ablation studies and the evaluation results are finally given to compare with state-of-the-art methods.

### Datasets

The experiments are conducted on different challenging datasets including the REFUGE dataset^1^ [83] and DRISHTI-GS datasets^2^ [84]. Specifically, the DRISHTI-GS dataset contains 101 images while the REFUGE dataset contains 1200 images. For the REFUGE dataset, we pre-train the network on the whole DRISHTI-GS dataset (101 images), REFUGE training and testing dataset (800 images) and evaluate the model on the REFUGE validation dataset (400 images). For the DRISHTI-GS dataset, it contains 101 retinal fundus images that 50 images are for training and 51 images are for testing. We pre-train the network on the DRISHTI-GS training set (50 images), the whole REFUGE datasets(1200 images) and finetune the pretrained on DRISHTI-GS training dataset(50 images), and finally evaluate the model on the DRISHTI-GS test dataset.

Note that the original images for the DRISHTI-GS dataset were provided by Aravind eye hospital, Madurai, who selected an approximately equal number of men and women, aged 40-80 years, with glaucoma and non-glaucoma patients for fundus image acquisition. All images were acquired with dilated pupils and captured according to the following data collec- tion protocol: OD-centred High-resolution fundus images of 2896 $\times$ 1944 pixels were acquired with a field of view of 30^∘^. Finally, by removing the surrounding non-fundus black area, the image area with the retinal structure is extracted from the original image, thereby obtaining a fundus image with a resolution of about 2047 $\times$ 1760. As shown in Fig. 2, each image was manually labelled by four glaucoma specialists with 3, 5, 9 and 20 years of experience, respectively. REFUGE dataset was organized as a half day Challenge in conjunction with the 5th MICCAI Workshop on Ophthalmic Medical Image Analysis (OMIA) with the goal of the challenge is to evaluate and compare automated algorithms for glaucoma detection and optic disc/cup segmentation on a common dataset of retinal fundus images. With this challenge, a large dataset of 1200 annotated retinal fundus images are made available. In addition, an evaluation framework has been designed to allow all the submitted results to be evaluated and compared with one another in a uniform manner. In general, these two datsets are currently the largest, most authoritative, and most challenging datasets. Therefore, we choose these two data sets to verify the effectiveness of our proposed network.

### Metircs

Following the previous works [8, 27] strictly, we evaluate the performance of the proposed method using the F1 score, Boundary distance Localization Error (BLE) and the Dice coefficients (DC). Among them, the definition of F1 can be computed as follows:

10 $$\begin{aligned} F1 = 2 \times \frac{\text {Precision} \times \text {Recall}}{\text {Precision} + \text {Recall}}, \end{aligned}$$

where Precision = $\frac{TP}{TP + FP}$ and Recall = $\frac{TP}{TP + FN}$. TP, TN, FP and FN represent true-positive, true-negative, false-positive and false-negative cases, respectively.

As for Dice coefficients (DC), it can be defined as follows:

11 $$\begin{aligned} DC = \frac{2 \times TP}{2 \times TP + FP + FN}. \end{aligned}$$

As for BLE, it can better reflect the segmentation effect of the boundary, which can be computed as follows:

12 $$\begin{aligned} BLE(C_0, C_g) = \frac{1}{N} \sum ^{N-1}_{\theta =0} \sqrt{(d^{\theta }_{g})^2 - (d^{\theta }_{0})^2}, \end{aligned}$$

where $d^{\theta }_{g}$ and $d^{\theta }_{0}$ indicate the Euclidean distance from the centre point of OD in the $\theta$ direction to $C_g$ and $C_0$, and 24 equidistant points (N = 24) are set in the evaluation. Note that the smaller the BLE, the better the segmentation effect.

### Implementations details

For supervised training, we train the entire network for 100 epochs. The learning rate decays 10 times every 50 epochs. The training is performed on one NVIDIA TITAN XP GPU. The initial learning rate is set to 0.0001. The batch size is set to 1. It takes almost 6 hours to train a network. For self-supervised pretraining, we train the network for 30 epochs with an initial learning rate of 0.0001 and decay 10 times every 15 epochs. The batch size is set to 8 on a single GPU since a bigger batch size may be hard to train and unstable as suggested by [69].

### Ablation study

To explore the components of our proposed method, we first conduct extensive analysis on DRISHTI-GS datasets [84] to demonstrate how they help to improve feature learning for optic disc and cup segmentation. Specifically, we will analyze the effect of the proposed multi-scale attention module, aggregation attention module, self-supervised learning strategy, and standard attention module, etc.

As shown in Table 2, we conduct plenty of ablative analysis of the proposed modules. Specifically, we can observe that our baseline model can only achieve acceptable but not competitive performance. When we inject the proposed multi-scale attention module, the segmentation performance of both the optic disc and cup can be improved by around 0.1 - 0.2 and 1.0 - 1.5 in terms of the F1 score and BLE metric. It is worth noting that we also conduct experiments using the standard attention block proposed in [22], as can be seen in the second row of Table 2. The results show that our proposed modified multi-scale attention module is better than the traditional one, which reveals the effectiveness of the proposed module. Furthermore, we explore the usefulness of the proposed aggregation attention module. Likewise, this component can also boost the network performance. In terms of self-supervised training, since we can use unlabeled data from other datasets for a large amount of unsupervised pre-training, we can first learn the encoder weights of a segmentation network with appropriate parameters. Then, we can fine-tune the entire segmentation network from the perspective of a global optimal solution. As can be seen in the fifth row of Table 2, self-supervised pre-training can benefit the segmentation performance by a large margin, which also demonstrates the necessity of self-supervised pre-training from unlabeled data. Ultimately, we have tried to combine the different proposed modules in pairs, and we can find that there are different levels of advanced improvements in this task. When we use our full model (all the proposed components are used), we can achieve the best performance.

**Table 2:** Analysis of the different proposed components on the DRISHTI-GS dataset

Method	OD		OC	
	*F1*	*BLE*	*F1*	*BLE*
Baseline	0.9334	9.07	0.8265	19.97
+ Standard Attention	0.9388	8.98	0.8314	19.19
+ Multi-Scale Attention	0.9488	8.17	0.8486	18.24
+ Aggregation Attention Module	0.9433	8.21	0.8432	18.47
+ Self-supervised Pretrain	0.9517	7.83	0.8636	15.72
+ Multi-Scale Attention + Aggregation Attention Module	0.9652	7.01	0.8879	13.98
+ Multi-Scale Attention + Self-supervised Pretrain	0.9788	6.28	0.9001	11.03
+ Aggregation Attention Module + Self-supervised Pretrain	0.9763	6.34	0.8978	11.54
Ours (full)	**0.9801**	**6.21**	**0.9087**	**10.07**

### Cross-validation

As shown in Table 3, we conduct a 5-fold verification experiment. Specifically, since the dataset itself is divided into a training set and a test set, here we divide the data set into 5 equal parts and conduct cross-validation experiments. It can be seen that the effect of our algorithm on each fold is relatively average, which also reflects the robustness and effectiveness of our algorithm.

**Table 3:** 5-fold Cross-validation on the DRISHTI-GS dataset

Method	OD		OC	
	*F1*	*BLE*	*F1*	*BLE*
1-fold	0.9801	6.21	0.9087	10.07
2-fold	0.9799	6.23	0.9066	10.12
3-fold	0.9800	6.19	0.9085	10.10
4-fold	0.9797	6.25	0.9083	10.11
5-fold	0.9798	6.24	0.9082	10.10

### Compare with the state-of-the-art methods

**Compared with DRISHTI-GS challenge**: To demonstrate the superiority of the proposed network, we compare the experimental results with the existing state-of-the-art segmentation methods, as shown in Table 4. We can observe that some previous representative works like FCN [32] and U-Net [12] networks fail to achieve satisfactory performance, whose F1 score and BLE metric are all below average. Although there were some improved methods later, such as POSAL [17], CE-Net [85] and JointRCNN [14]. Most of these methods only focus on how to improve the design of the model artificially and do not take into account the scarcity of medical data and the expansion of the convolutional receptive field. These methods are easily interfered by fundus blood vessels and can not segment the boundary contour well. Our designed method comprehensively considers the existing segmentation problems from both the data and model perspectives, and we can finally achieve the best performance over the previous methods. Besides, the parameter of our network is also competitive, which guarantees the effectiveness of execution speed.

**Table 4:** Comparison of quantitative results of different methods on the DRISHTI-GS dataset. Some of the results are derived from [8]

Method	Year	Params (M)	FLOPs (G)	Times (ms)	OD		OC	
					*F1*	*BLE*	*F1*	*BLE*
FCN [32]	2014	48.2	136.2	500	0.9321	8.90	0.8170	21.83
U-Net [12]	2015	65.9	158.3	623	0.9600	7.23	0.8500	19.53
M-Net [13]	2018	71.8	164.5	650	0.9590	7.97	0.866	17.05
Stack-U-Net [86]	2018	86.6	178.0	700	0.9700	6.47	0.8900	14.39
POSAL [17]	2019	68.5	-	-	0.9650	-	0.8580	-
CE-Net [85]	2019	71.3	125.0	550	0.9688	5.04	0.8699	16.06
JointRCNN [14]	2020	-	-	-	0.9640	-	0.8640	-
BGA-Net [87]	2021	75.6	148.5	600	0.9750	7.01	0.8980	14.37
DCGAN [88]	2022	102.9	-	-	0.9746	7.35	0.8631	18.69
RSAP-Net [8]	2022	87.6	235.0	800	0.9752	6.33	0.9012	11.97
Ours	2023	54.6	147.6	560	**0.9801**	**6.21**	**0.9087**	**10.07**

**Compared with REFUGE challenge**: We also compare our segmentation results with state-of-the-art methods on the REFUGE challenge task. As shown in Table 5, the first 12 rows are the results from different participating teams, and the rest are the results of some publicly available deep learning methods. Notably, our method can achieve the best segmentation result on both the optic disc and cup. The performance on both the REFUGE dataset and DRISHTI-GS datasets all reflect the generalizability and feasibility of the proposed network and training paradigm.

**Table 5:** Comparison of quantitative results of different methods on the REFUGE dataset. Some of the results are derived from [27]

Method	DC_disc_	DC_cup_
CHUKMED	0.9602	0.8826
Masker	0.9496	0.8837
BUCT	0.9525	0.8728
NKSG	0.9488	0.8643
VRT	0.9532	0.8600
AIML	0.9505	0.8519
Mammoth	0.9361	0.8667
SMILEDeepDR	0.9386	0.8367
NightOwl	0.9487	0.8257
SDSAIRC	0.9436	0.8315
Cvblab	0.9077	0.7728
WinterFell	0.8772	0.6861
M-Net [13]	0.9436	0.8315
POSAL [17]	0.9602	0.8826
Mask R-CNN [82]	0.9504	0.8546
Liu et al. [27]	0.9601	0.8903
Ours	**0.9657**	**0.8976**

**Fig. 7:**
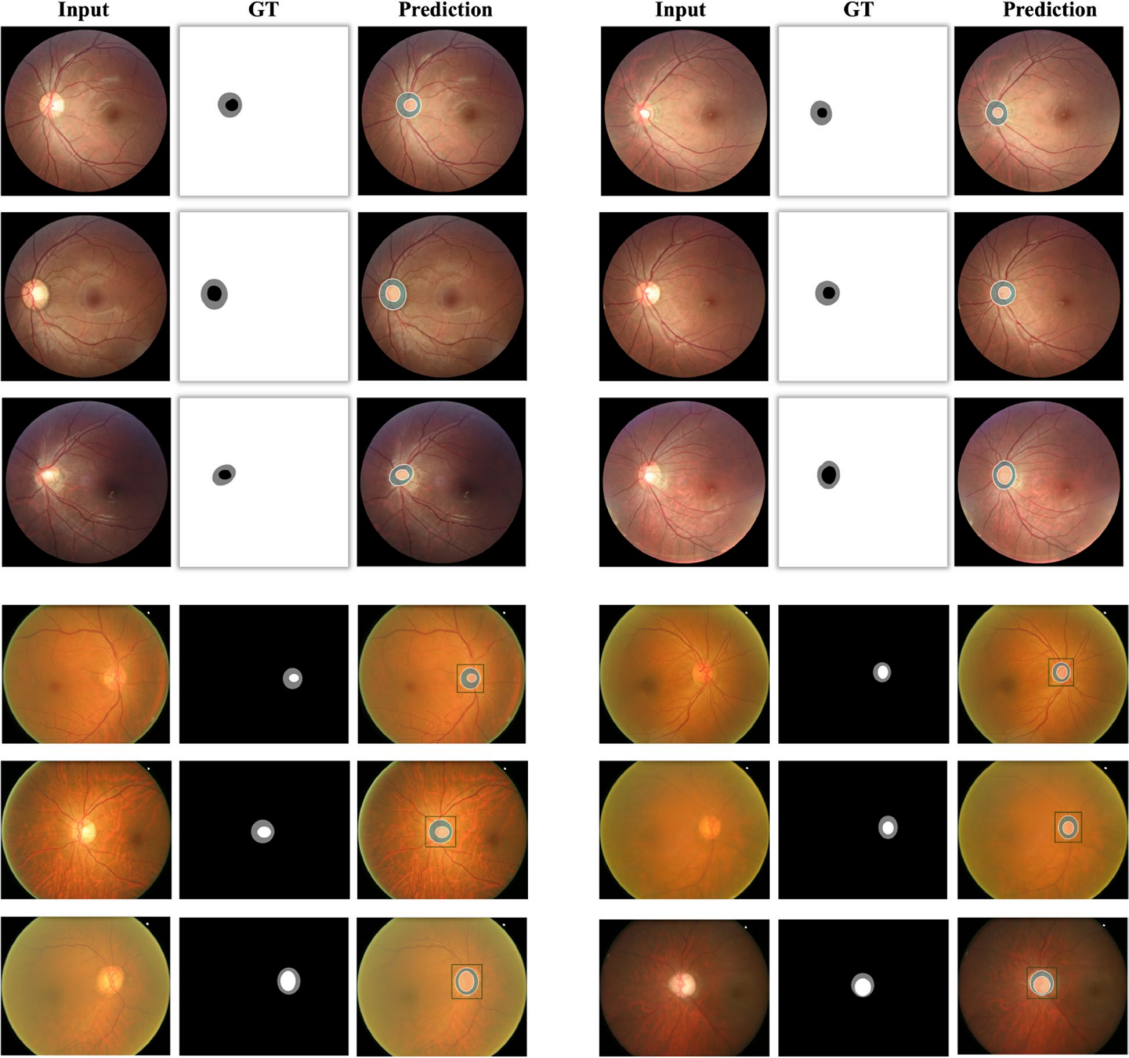
Visualizations of the optic disc and cup segmentation on REFUGE dataset and DRISHTI-GS dataset

**Qualitative visualization**: Fig. 7 shows some qualitative visualizations of our proposed method on both the REFUGE dataset and DRISHTI-GS datasets. As can be seen, our method can yield high-quality accurate masks, which demonstrates that our method can be applied to practical medical applications.

**Computational Complexity Analysis**: As shown in Table 4, we also provide the computational complexity of different state-of-the-art networks, including network parameters, floating-point operations per second (GFLOPs) and running time. We can observe that although some of the previous CNN-based networks enjoyed low computational complexity, they failed to achieve satisfactory performance. The proposed framework can make a good balance between network performance and computational complexity.

**Discussion**: Through systematic experiments and evaluations such as the qualitative comparative experiments shown in Tables 4 and 5, we can see that our method has more advantages than existing advanced methods. We believe there are the following reasons: (1) First, we make an early attempt to adopt an unsupervised pre-training strategy, which can use a large amount of unlabeled data for image representation learning so that the network can be optimized in a better direction; (2) The proposed attention mechanism can effectively help the network expand the receptive field of learning, allowing the network to learn the global information of medical images and effectively improve the segmentation effect. Overall, the network we designed can efficiently solve the current joint optic disc and cup segmentation tasks.

**Limitation**: As a common practice in the deep learning area, every framework will have certain limitations. Among them, we generously admit that our method will be somewhat cumbersome in terms of training time because it is trained in two steps (i.e., self-supervised pre- train and then combined with supervised training). However, we believe that self-supervised training is a new training strategy that does not increase the number of parameters of the network operation. In addition, because we use the transformer-based attention mechanism, this will cause our network to be more computationally intensive than traditional CNN-based networks. However, the current GPU acceleration technique can already solve these problems well.

**Future Work**: In future work, we will continue to explore various variants of attention mechanism structures, hoping to effectively solve specific problems in the field of medical images. At the same time, we will also focus more on designing lightweight networks to be more suitable for practical applications in the medical field. Finally, we will also focus on other optimization methods in the field of self-supervised learning to solve problems such as training model collapse and parameter sensitivity and use the capabilities of large models to solve some issues such as data imbalance and data migration.

## Conclusion

In this paper, we deeply discuss and analyze the unresolved challenges in medical segmentation especially for optic disc and cup segmentation. We then propose a novel attention-aware encoder-decoder network equipped with the designed multi-scale attention block and the aggregation attention module, which is capable of helping the network to capture the global dependencies of the input image tokens. Furthermore, we introduce a novel loss function to make use of the knowledge by constraining the subtraction of the optic cup from the optic disc in the optic rim and adopt contrastive learning for self-supervised pre-training. This strategy can alleviate the shortcomings of a small amount of image training data in the medical field. Finally, extensive experimental results conducted on different challenging benchmarks all demonstrate the superiority of the proposed network and training paradigm, which can outperform other state-of-the-art methods.

## Data Availability

The data presented in this study are available on request from the corresponding author.

## Footnotes

1 https://refuge.grand-challenge.org/Home2020/

2 https://www.kaggle.com/datasets/lokeshsaipureddi/drishtigs-retina-dataset-for-onh-segmentation
